# Tunable and Anisotropic Dual-Band Metamaterial Absorber Using Elliptical Graphene-Black Phosphorus Pairs

**DOI:** 10.1186/s11671-019-3182-9

**Published:** 2019-11-21

**Authors:** Yijun Cai, Shuangluan Li, Yuanguo Zhou, Xuanyu Wang, Kai-Da Xu, Rongrong Guo, William T. Joines

**Affiliations:** 1 Fujian Provincial Key Laboratory of Optoelectronic Technology and Devices, Xiamen University of Technology, Xiamen, 361024 China; 2 College of Communication and Information Engineering, Xi’an University of Science and Technology, Xi’an, 710054 China; 3 Department of Electrical and Computer Engineering, University of Wisconsin–Madison, Madison, WI 53706 USA; 4 Department of Electrical and Computer Engineering, Duke University, Durham, NC 27708 USA

**Keywords:** Metamaterial absorber, Two-dimensional material, Dual-band absorber, Surface plasmons

## Abstract

We numerically propose a dual-band absorber in the infrared region based on periodic elliptical graphene-black phosphorus (BP) pairs. The proposed absorber exhibits near-unity anisotropic absorption for both resonances due to the combination of graphene and BP. Each of the resonances is independently tunable via adjusting the geometric parameters. Besides, doping levels of graphene and BP can also tune resonant properties effectively. By analyzing the electric field distributions, surface plasmon resonances are observed in the graphene-BP ellipses, contributing to the strong and anisotropic plasmonic response. Moreover, the robustness for incident angles and polarization sensitivity are also illustrated.

## Introduction

Graphene is a two-dimensional material with carbon atoms arranged in a honeycomb lattice [1, 2]. Various graphene-based photonic devices have been developed in the recent years due to their ultracompact size and unique light-graphene interaction [3–6]. As one of its most significant applications, metamaterial absorbers based on graphene have attracted burgeoning amount of interest due to their strong and tunable plasmonic response [7–10]. However, several applications that require high on-off ratio are restricted due to the zero or near-zero band gap of graphene [11]. As an alternative two-dimensional material, black phosphorus (BP), a monolayer of phosphorus atoms arranged in a hexagonal lattice with a puckered structure [12], has also received a surge of research interest recently. It possesses exceptional optical and electronic properties, such as in-plane anisotropy, thickness-dependent tunable band gap [13], and high carrier density and mobility [14]. Over the past few years, in the infrared region, researchers have investigated numerous structures to enhance the light-BP interaction strength in the metamaterial based on BP [15–17]. Nevertheless, the plasmonic resonance of BP-based absorber is hardly to be tuned flexibly and effectively, and they normally suffer from relatively low absorption rate with moderate doping level. This is attributed to the fact that the resonance strength in monolayer BP is rather weak, limiting its anisotropic potentials. Thus, graphene-BP-based plasmonic absorbers have been proposed utilizing the hybridization of graphene and BP to achieve strong and anisotropic plasmonic absorption [18–20]. However, the previous reported graphene-BP-based absorbers generally require relatively complicated fabrication technique or possess single absorption band, impeding their further applications for imaging, biosensing, and communication systems.

In our work, an anisotropic dual-band infrared absorber is numerically proposed using periodic elliptical graphene-BP pairs, which is ease of fabrication. The independent tunability of resonance by geometric size and doping level is demonstrated. Electric field distributions are plotted to reveal the physical mechanism. The incident angle tolerance and polarization sensitivity are also illustrated.

## Methods

**Fig. 1:**
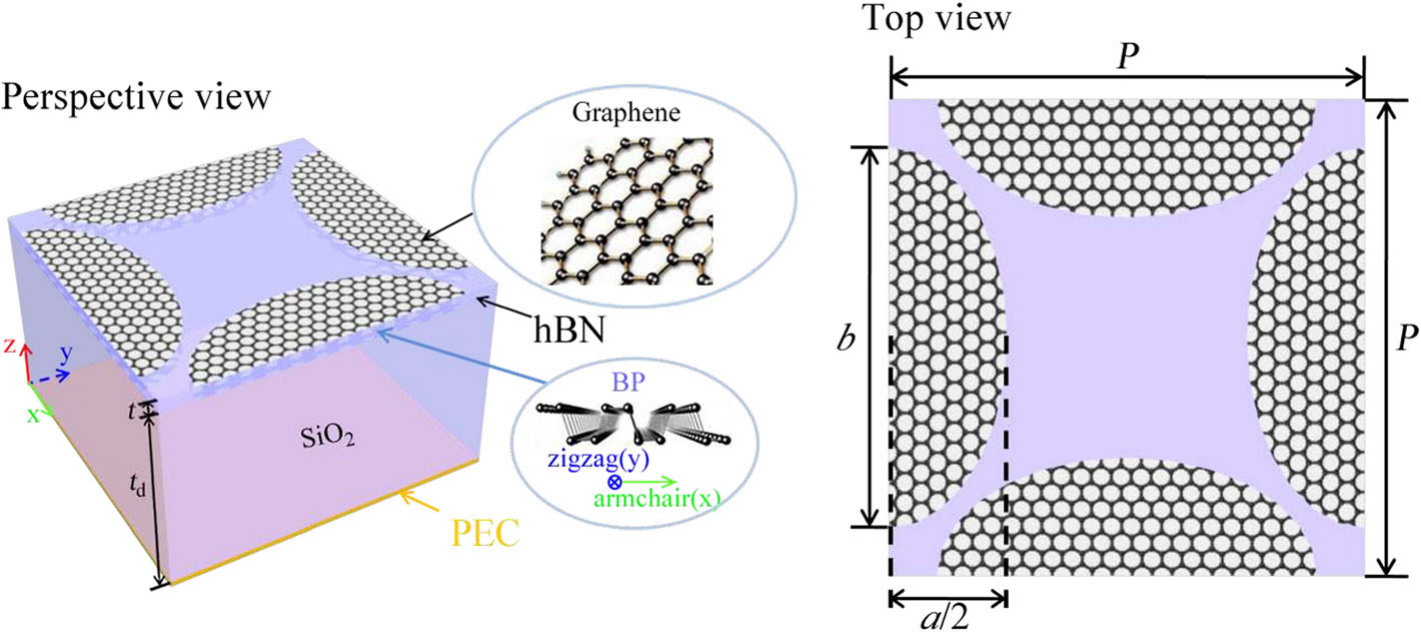
A unit cell of the proposed absorber based on elliptical graphene-BP pairs. *t*_d_ and t are the thicknesses of the dielectric and insulator layer, respectively. *a* and *b* are the short axis and long axis of the ellipse. *P* is the periodic side length of the square unit cell

The proposed absorber is made up of transverse and longitudinal elliptical graphene-BP pairs deposited on a SiO_2_ layer as shown in Fig. 1. A hexagonal boron nitride (hBN) layer is inserted between monolayer graphene and BP as an insulating spacer to prevent carrier transport between them and guarantee high carrier mobility. The parameters of SiO_2_ and hBN are obtained from Ref. 21 and Ref. 22 respectively. The simulations are carried out by COMSOL Multiphysics to investigate the dual-band properties, which is based on finite element method (FEM) in the frequency domain. We apply Floquet periodicity as the boundary conditions in both *x*- and *y*- directions. A port with infrared wave excitation is set upon the top surface of the computational domain, while perfect electric conductor (PEC) boundary condition is set on the bottom surface. Tetrahedral meshes with user-controller mesh density are applied for the entire domain.

In the simulation, both graphene and BP are treated as two-dimensional surface with surface conductivities instead of bulk materials with permittivity tensors. This assumption solves the problems of thickness definition for ultrathin materials and low computational efficiency [23].

To describe the surface conductivity of graphene *σ*(*ω*), we use the well-known Kubo formulas as below [24]:

1 $$ \sigma \left(\omega, {\mu}_c,\varGamma, T\right)={\sigma}_{\mathrm{intra}}+{\sigma}_{\mathrm{inter}} $$

2 $$ {\displaystyle \begin{array}{l}{\sigma}_{\mathrm{intra}}=\frac{j{e}^2}{\pi {\hslash}^2\left(\omega -j2\varGamma \right)}\\ {}\kern2em \times {\int}_0^{\infty}\xi \left(\frac{\partial {f}_d\left(\xi, {\mu}_c,T\right)}{\partial \xi }-\frac{\partial {f}_d\left(-\xi, {\mu}_c,T\right)}{\partial \xi}\right) d\xi\ \end{array}} $$

3 $$ {\displaystyle \begin{array}{l}{\sigma}_{\mathrm{inter}}=-\frac{j{e}^2\left(\omega -j2\varGamma \right)}{\pi {\hslash}^2}\\ {}\kern2.25em \times {\int}_0^{\infty}\frac{f_d\left(-\xi, {\mu}_c,T\right)-{f}_d\left(\xi, {\mu}_c,T\right)}{{\left(\omega -j2\varGamma \right)}^2-4{\left(\xi /\hslash \right)}^2} d\xi \end{array}} $$

4 $$ {f}_d\left(\xi, {\mu}_c,T\right)={\left({e}^{\left(\xi -{\mu}_c\right)/{k}_BT}+1\right)}^{-1} $$

According to Eq. 1, *σ*(*ω*) consists of the intraband and interband counterparts, namely *σ*_intra_ and *σ*_inter_. *ω* is the radian frequency, μ_c_ is the chemical potential, *Г* is the scattering rate, and *T* is the Kelvin temperature. *ħ*, *e*, *ξ*, and *k*_*B*_ are the reduced Planck constant, electron charge, electron energy, and Boltzmann constant, respectively.

In the infrared region, since the incident photon can hardly excite the interband transition, the light-graphene interaction is dominated by the intraband transition. Particularly, when μ_c_ ≫ *k*_B_*T*, Kubo formulas can be further simplified to Eq. 5:

5 $$ {\sigma}_g=\frac{i{e}^2{\mu}_c}{\pi {\hslash}^2\left(\omega +i2\varGamma \right)} $$

Thus, the surface conductivity of graphene is dependent on the values of *ω*, *Г*, and *μ*_*c*_. Here, *Г* is assumed as 0.3 meV and *μ*_c_ is assumed to be 0.7 eV according to the previous work [25, 26].

On the other hand, we calculate the surface conductivity *σ*_j_ of BP with a simple semi-classical Drude model [27]:

6 $$ {\sigma}_j=\frac{iD}{\pi \left(\omega +\frac{i{\varGamma}_{\mathrm{BP}}}{\hslash}\right)} $$

7 $$ {D}_j=\frac{\pi {e}^2{n}_s}{m_j} $$

where *n*_s_ is the carrier density relating with the doping level. We choose *n*_s_ = 1.9 × 10^13^ cm^−2^ and *Г*_*BP*_ *=* 10 meV according to the previous reference [16]. *j* is the concerned direction, so *σ*_*x*_ and *σ*_*y*_ are determined by the electron mass along *x*- and *y*-direction, respectively. *m*_x_ and *m*_y_ can be further calculated by:

8 $$ {m}_x=\frac{\hslash^2}{\frac{2{\gamma}^2}{\varDelta }+{\eta}_c} $$

9 $$ {m}_y=\frac{\hslash^2}{2{\nu}_c} $$

10 $$ {\eta}_c=\frac{\hslash^2}{0.4{m}_0} $$

11 $$ {v}_c=\frac{\hslash^2}{1.4{m}_0} $$

12 $$ \gamma =\frac{4a}{\pi } $$

where *m*_0_ is the standard electron mass, and *Δ* and *a* are the band gap and scale length for BP monolayer, respectively. By substituting Eqs. 10–12 into Eq. 8 and Eq. 9, one can obtain the electron mass along armchair (*x*-) and zigzag (*y*-) direction. The discrepancy between them contributes to the anisotropic surface conductivity of BP.

## Results and Discussion

**Fig. 2:**
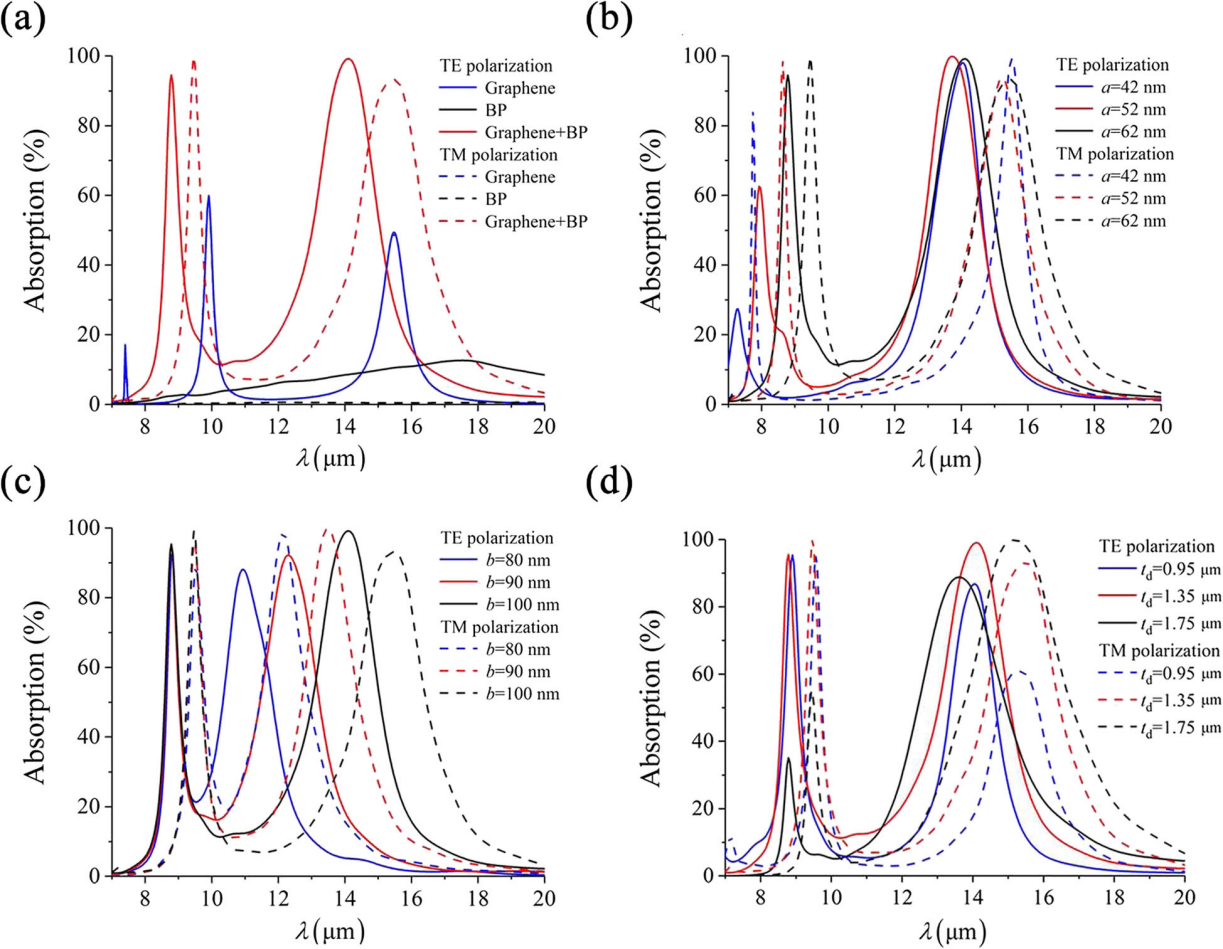
**a** Comparison of plasmonic responses between monolayer graphene (blue solid curve and blue dashed curve are overlapped), monolayer BP and graphene-BP pairs, and absorption spectra with different *a* (**b**), *b* (**c**), and *t*_d_ (**d**). The default parameters are *a* = 62 nm, *b* = 100 nm, *t*_d_ = 1.35 μm, *t* = 5 nm, and *P* = 250 nm, under normal incidence

To illustrate the anisotropic absorption characteristic of the proposed absorber, we first simulate and compare the absorption spectra with individual graphene layer, individual BP layer, and graphene-BP pairs. As can been observed in Fig. 2a, the plasmonic response of graphene is isotropic with two obvious absorption peaks at 9.9 μm and 15.4 μm, independent on the polarization. On the other hand, although the plasmon resonance of BP is anisotropic, its strength is quite weak for either TE (< 12.7%) or TM (< 0.7%) incidence. By combining the advantages of graphene and BP, graphene-BP pairs exhibit both strong and anisotropic plasmonic responses. For TE incidence, the two absorption peaks are located at 8.8 μm and 14.1 μm, with absorption rates larger than 90%. For TM incidence, the wavelengths of maximum absorption are shifted to 9.5 μm and 15.4 μm, respectively. The polarization extinction ratio can be defined as PER = 10 × log(*R*_1_/*R*_0_), where *R*_1_ and *R*_0_ denote the reflectance (*R* = 1-*A*, *A* represents the absorbance) of different polarizations at the same wavelength, then the maximum PER of each resonance can reach up to 23 dB and 25 dB at *λ* = 9.5 μm and *λ* = 14.1 μm, respectively. Therefore, the proposed absorber can be utilized as a dual-band reflective polarizer with high performance.

We next analyze the absorption spectra with different geometric configurations to demonstrate the tunable dual-band absorption property in Fig. 2b–d. In Fig. 2b, the first absorption peaks have redshifts as *a* increases from 42 to 52 nm for both polarizations, while second resonant frequencies are almost unchanged. On the other hand, as shown in Fig. 2c, by increasing the long axis length *b*, the second resonances are redshifted as well, while the first absorption peaks remain constant for TE and TM polarization. Therefore, the dual absorption peaks can be tuned independently by varying the corresponding axis length in the elliptical graphene-BP pairs. Moreover, the thickness of dielectric layer also plays a critical role in the performance for the proposed device, which acts as a Fabry-Perot resonator formed by the graphene-BP metasurface and the PEC substrate. Thus, the absorption spectra with different *t*_d_ are plotted in Fig. 2d. As *t*_d_ increases from 0.95 to 1.75 μm, the first absorption peaks for TE and TM polarization have a dramatic drop, while the second peaks increase at first then decrease sharply. As a consequence, there is an optimal thickness *t*_d_ that maximizes the dual absorption peaks of the proposed absorber.

**Fig. 3:**
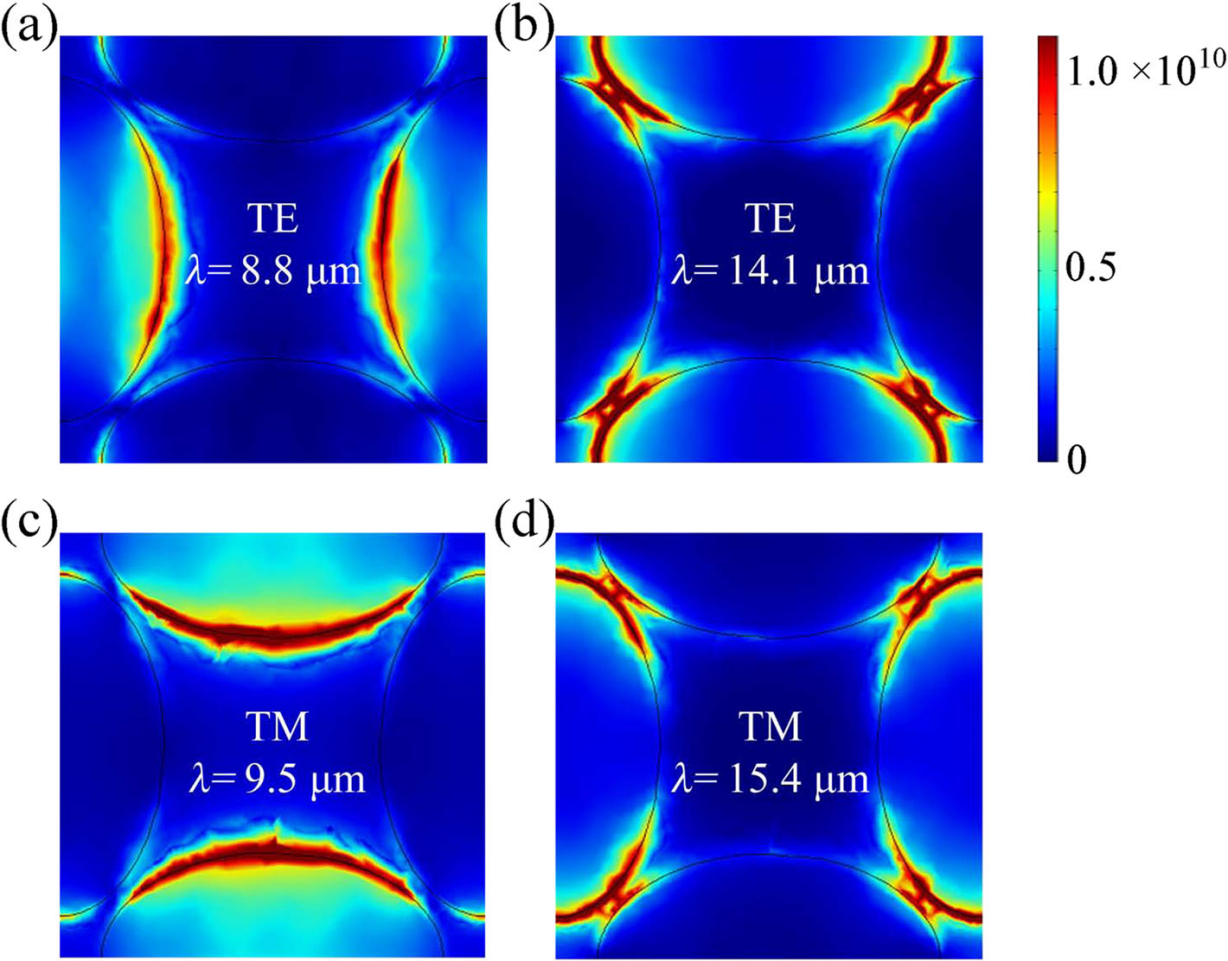
Electric field intensity distributions at different wavelengths for **a**, **b** TE and **c**, **d** TM polarization, where *a* = 62 nm, *b* = 100 nm, *t*_d_ = 1.35 μm, *t* = 5 nm, *P* = 250 nm, under normal incidence

In order to elucidate the physical insight, we further reveal the electric field intensity distributions at different wavelengths in Fig. 3. For TE incidence, the electric field is in the armchair (*x*-) direction. At the first peak (*λ* = 8.8 μm), the incident infrared light can excite electrons in graphene and BP to oscillate in the transverse direction, leading to the concentration of electric field at the short axis ends of the longitudinal ellipse as shown in Fig. 3a. At *λ* = 14.1 μm, the localized electric field is enhanced at the long axis ends of the transverse ellipse. On the other hand, TM incidence with electric field in the zigzag (*y*-) direction can excite electrons to vibrate along the longitudinal direction at the absorption peak of 9.5 μm, leading to concentrated field distributions at the short axis ends of the transverse ellipse. Besides, at *λ* = 15.4 μm, the enhancement of electric field is focused at the long axis ends of the longitudinal ellipse. Therefore, the resonance wavelengths are directly related to the finite oscillation length of the induced dipoles in both transverse and longitudinal elliptical graphene and BP pairs.

**Fig. 4:**
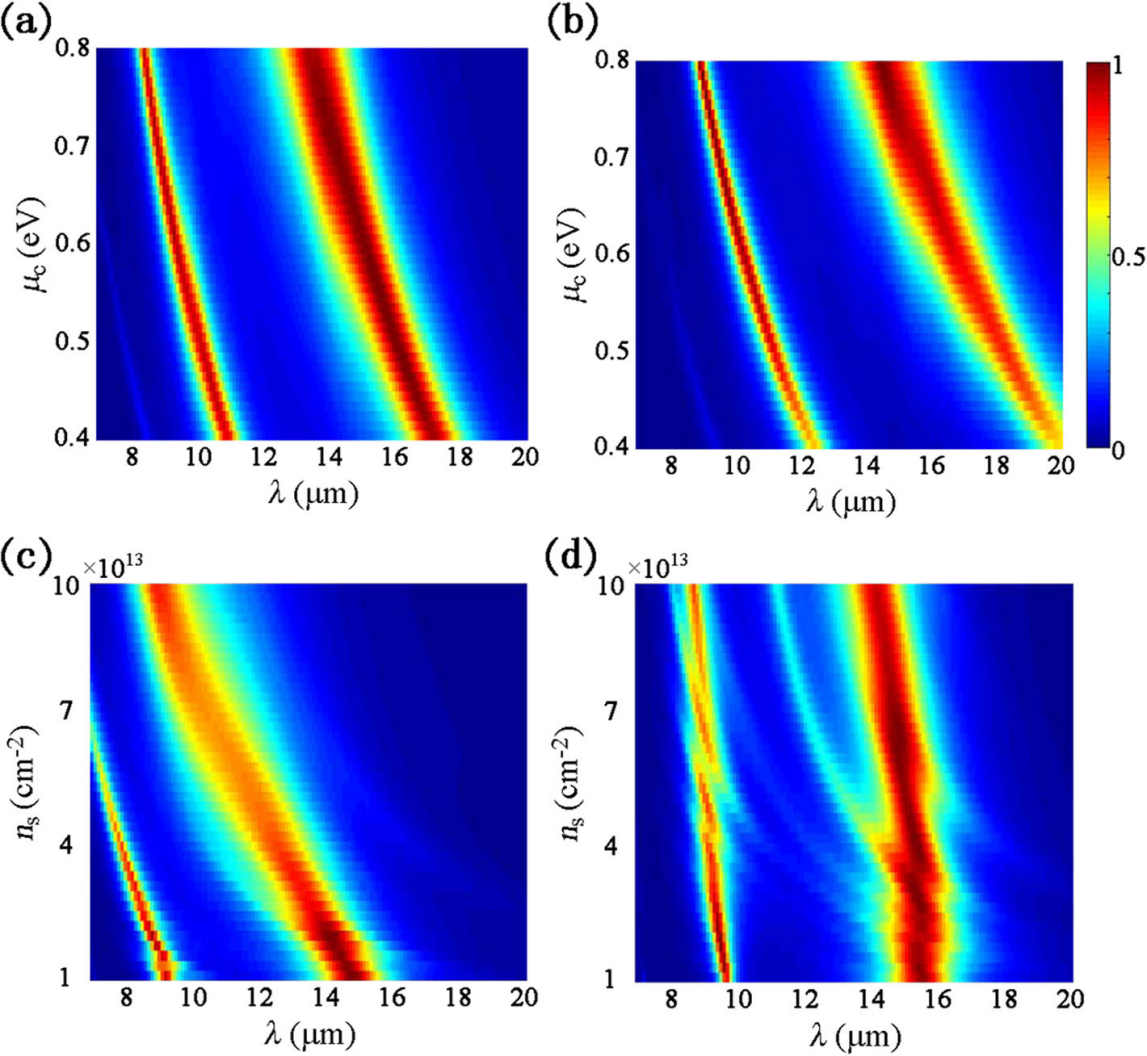
Absorption spectra versus different doping levels under normal incidence: **a** and **b** for varied chemical potentials of graphene, **c** and **d** for varied carrier densities of BP, **a** and **c** for TE polarization, and **b** and **d** for TM polarization, where *a* = 62 nm, *b* = 100 nm, *t*_d_ = 1.35 μm, *t* = 5 nm, and *P* = 250 nm

One can tune the anisotropic dual-band absorption performance effectively by varying the geometric dimensions as demonstrated in Fig. 2b–d. Meanwhile, the surface conductivities of graphene and BP can also be manipulated by varying μ_c_ and *n*_s_ according to graphene and BP model formulas as mentioned above. *μ*_c_ and *n*_s_ represent the doping level of graphene and BP that can be altered after geometric fabrication. Thus, performances of the proposed absorber with different *μ*_c_ and *n*_s_ are depicted in Fig. 4. Considering the practical situation, *μ*_c_ is chosen between 0.4 and 0.8 eV from the previous work verified by experiments [28]. In the previous reported work [29], the maximum theoretical value for *n*_s_ of BP was demonstrated to be 2.6 × 10^14^ cm^−2^, so a moderate *n*_s_ is chosen between 10^13^ cm^−2^ and 10^14^ cm^−2^ in the simulation. In Fig. 4a, when *μ*_c_ = 0.4 eV, the first absorption peak is located at 10.9 μm and the second one is located at 17.1 μm. As *μ*_c_ increases to 0.8 eV, the two resonant wavelengths are blueshifted to 8.4 μm and 13.4 μm. Similarly for TM polarization, the dual absorption peaks are blueshifted from 12.4 and 19.8 μm to 8.9 and 14.4 μm, respectively, with *μ*_c_ increasing from 0.4 to 0.8 eV as shown in Fig. 4b. For individual patterned BP, the resonance wavelength *λ*_p_ can be calculated as ${\lambda}_p\propto \sqrt{L/{n}_s}$, where *L* is the effective oscillation length [27]. Thus, if *L* is fixed, the absorption spectra exhibit an obvious blueshift as *n*_s_ increases for TE polarization as plotted in Fig. 4c. For TM polarization, the absorption peaks are also slightly blueshifted as *n*_s_ increases from 10^13^ cm^−2^ to 10^14^ cm^−2^ as demonstrated in Fig. 4d.

**Fig. 5:**
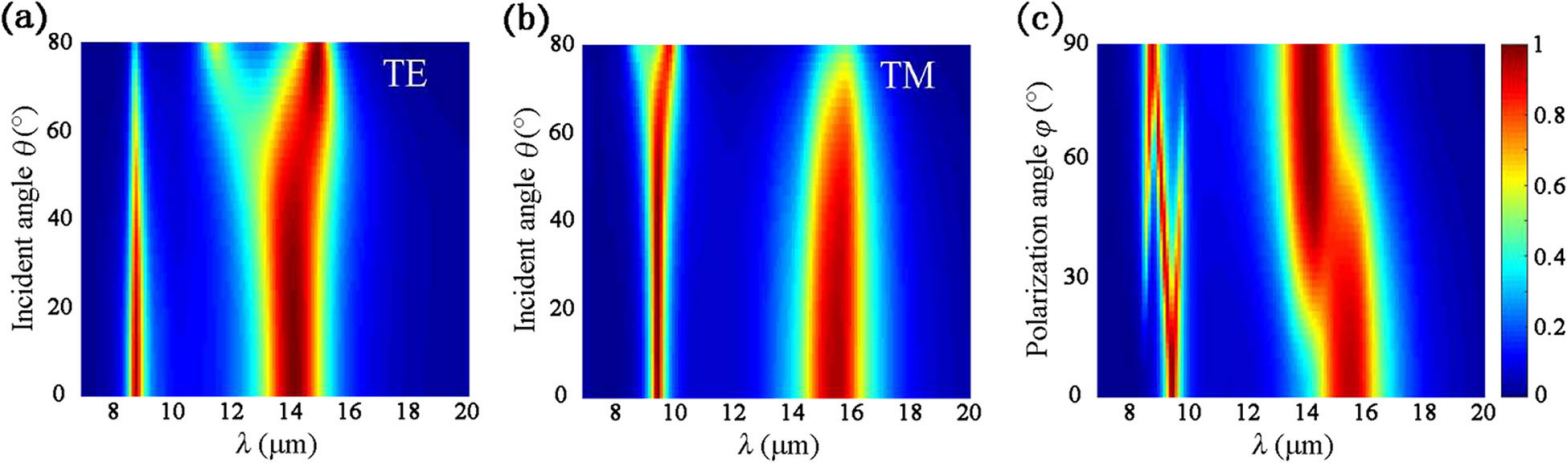
Absorption spectra under various incident angles for **a** TE and **b** TM polarization and **c** various polarization angles under normal incidence. Geometric parameters are the same as in Fig. 4

In the practical applications, tolerance of wide incident angles is preferred for infrared absorbers. Therefore, absorption spectra under oblique incidences are elaborated. In Fig. 5a, it is observed that, for TE polarization, the first absorption peak remains larger than 80% when *θ* increases to 52°, while the second absorption peak maintains above 80% even when *θ* increases to 80°. When *θ* > 46°, the second resonant wavelength is redshifted gradually as *θ* becomes larger. For TM incidence, when *θ* is less than 62°, the absorption rate at the first peak maintains larger than 90%, while the resonant wavelength keeps constant at *λ* = 9.5 μm as shown in Fig. 5b. Besides, for the second resonance, the peak absorption remains larger than 80% with *θ* up to 60°, then drops slightly with the increase of *θ*. The excellent angular stability originates from the common feature of Fabry-Perot resonators, which are robust for oblique incident angles [30].

Absorption spectra under normal incidence with different polarization angles *φ* are presented in Fig. 5c to investigate the polarization dependence of the proposed absorber. We assume the polarization angle of TE polarization to be 0°. One can see from Fig. 5c that, as *φ* increases from 0 to 90°, the absorption spectrum turns out to be the same as the TM polarization in Fig. 2a. When 0° < *φ* < 90°, the incidence will excite electrons in BP to oscillate in both armchair and zigzag directions due to its *x*- and *y*- components of the incident electric field. Consequently, surface plasmon resonances can be induced simultaneously in armchair and zigzag directions of BP.

## Conclusions

In conclusions, we have proposed an anisotropic dual-band infrared absorber consisting of periodic transverse and longitudinal graphene-BP ellipses. The maximum PER at each resonance can reach up to 23 dB and 25 dB. The dual anisotropic resonances are attributed to the induced electric dipoles located at the ends of short and long axes. By adjusting the lengths of short axis and long axis, the first and second absorption peaks can be independently tuned, respectively. Moreover, the resonant absorption bands can also be tuned by changing the corresponding doping level of graphene and BP. Besides, high absorption rates at both peaks can be achieved under oblique incidence for any polarization. The proposed absorber can be utilized as a tunable reflective polarizer and novel infrared sensor.

## Data Availability

All data are fully available without restriction.

## Abbreviations

BP: Black phosphorus
FEM: Finite element method
hBN: Hexagonal boron nitride
PEC: Perfect electric conductor
TE: Transverse electric
TM: Transverse magnetic
